# Patient Prosthesis Mismatch After SAVR and TAVR

**DOI:** 10.3389/fcvm.2022.761917

**Published:** 2022-03-30

**Authors:** Sabine Bleiziffer, Tanja K. Rudolph

**Affiliations:** ^1^Department of Thoracic and Cardiovascular Surgery, Heart and Diabetes Center North Rhine-Westphalia, University Hospital Ruhr-University Bochum, Bad Oeynhausen, Germany; ^2^Department for General and Interventional Cardiology/Angiology, Heart and Diabetes Center North Rhine-Westphalia Bochum, University Hospital of the Ruhr University, Bad Oeynhausen, Germany

**Keywords:** aortic valve stenosis, prosthesis-patient mismatch (PPM), SAVR valves, TAVR - outcomes and related issues, effective orifice area (EOA)

## Abstract

Patient-prosthesis mismatch (PPM) remains one out of many factors to be considered during decision-making for the treatment of aortic valve pathologies. The idea of adequate sizing of a prosthetic heart valve was established by Rahimtoola already in 1978. In this article, the author described the phenomenon that the orifice area of a prosthetic heart valve may be too small for the individual patient. PPM is assessed by measurement or projection of the prosthetic effective orifice area indexed to body surface area (iEOA), while it is recommended to use different cut point values for non-obese and obese patients for the categorization of moderate and severe PPM. Several factors influence the accuracy of both the projected and the measured iEOA for PPM assessment, which leads to a certain number of false assignments to the PPM or no PPM group. Despite divergent findings on the impact of PPM on clinical outcomes, there is consensus that PPM should be avoided to prevent sequelae of increased prosthetic gradients after aortic valve replacement. To prevent PPM, it is required to anticipate the iEOA of the prosthesis prior to the procedure. The use of adequate reference tables, derived from echocardiographically measured mean effective orifice area (EOA) values from preferably large numbers of patients, is most appropriate to predict the iEOA. Such tables should be used also for transcatheter heart valves in the future. During the decision-making process, all available options should be taken into account for the individual patient. If the predicted size and type of a surgical prosthesis cannot be implanted, additional surgical procedures, such as annular enlargement with the Manougian technique, or alternative procedures, such as transcatheter aortic valve implantation (TAVI) can prevent PPM. PPM prevention for TAVI patients is a new field of interest and includes anticipation of the iEOA, prosthesis selection, and procedural strategies.

## Introduction

The idea of adequate sizing of a prosthetic heart valve was established by Rahimtoola who published “The problem of valve prosthesis-patient mismatch” in Circulation in 1978 (1). In this article, the author described the phenomenon that the orifice area of a prosthetic heart valve may be too small for the individual patient. The topic is of continued interest until today. A substantial amount of data and reviews have been published to categorize and standardize definitions and assessment methods for prosthesis-patient mismatch (PPM) after surgical aortic valve replacement (SAVR). The question concerning the clinical impact of PPM is a matter of ongoing controversy. With the start of transcatheter aortic valve implantations (TAVIs) several new aspects emerged: does PPM also occur after TAVI? Is assessment different than after SAVR? Are there differences comparing SAVR and TAVI? What is the impact of PPM after SAVR and TAVI today and for future patient populations with expanding TAVI indications?

The aim of this review is to summarize the most-updated evidence about PPM after aortic valve replacement from the perspective of both surgeons and interventional cardiologists.

## PPM Measurement and Definition

Derived from case descriptions, Rahimtoola already stated in 1978 that the minimum prosthetic valve size which is required to avoid mismatch must be corrected to the body size of patient which reflects the hemodynamic requirements (1).

JG Dumesnil and P Pibarot further studied the question how to measure PPM adequately. Among the options to define the opening area of a prosthetic aortic valve, the effective orifice area (EOA) which is supposed to reflect the area available for blood flow indexed to body size, was considered most reliable (2, 3). Although being *per se* a continuous variable, it has been most practicable to categorize indexed effective orifice area (iEOA) into moderate and severe PPM (Table 1).

**Table 1:** Thresholds for prosthesis-patient mismatch (PPM).

	**Indexed EOA**		
	**No/mild PPM**	**Moderate PPM**	**Severe PPM**
Normal weight patients	≤ 0.65 cm^2^/m^2^	>0.65–0.85 cm^2^/m^2^	>0.85 cm^2^/m^2^
Obese patients (BMI ≥ 30 kg/m^2^)	≤ 0.55 cm^2^/m^2^	>0.55–0.7 cm^2^/m^2^	>0.7 cm^2^/m^2^

*BMI, body mass index; EOA, effective orifice area; PPM, patient-prosthesis mismatch*.

Other options to describe the valve opening size, such as the geometric prosthetic valve area, label size, or *in vitro* measurements could not show consistent prediction of or relation to clinical outcomes (4, 5). Geometric valve dimensions and *in vitro* measurements do not take into account the variations in the relative opening of the leaflets in relation to the balance between their resistive properties and the impetus provided by left ventricular outflow (6). The valve size labels of different manufacturers refer to different components of the prostheses and are not comparable. For example, the true inner diameter of a 23 mm labeled prosthesis can be 21 mm for the Perimount prosthesis, or 18.5 mm for the Hancock II prosthesis (7). To establish uniformity for providing prosthetic heart valve physical dimensions, the European Association for Cardio-Thoracic Surgery (EACTS), The Society of Thoracic Surgeons (STS), and American Association for Thoracic Surgery (AATS) set up a Task Force comprised of cardiac surgeons, cardiologists, engineers, regulatory bodies, representatives of the International Organization for Standardization, and major valve manufacturers. Their expert consensus document contains recommendations for the establishment of uniform, standardized charts to provide surgical heart valve dimensions, implant positions, and hemodynamic performance for all types of valve prostheses (8). Accordingly, it is to expect that the prosthetic heart valve choice can be based on objective, distinguishable reference numbers in the future.

It is routine to index the EOA to the body surface area (BSA). The question if BSA is the best reference for body size emerges particularly when comparing athletic and obese constitutions, because of different hemodynamic requirements. Obese patients might require less iEOA for a normal valve function, because cardiac output and stroke volume are more strongly related to fat-free body mass than adipose mass (9). It has been shown that PPM has less impact on clinical outcomes in adipose patients (10, 11). Therefore, it is recommended to use lower cut point values to define moderate and severe PPM in obese patients (12) (Table 1). The use of fat-free mass has not become routine.

Recently, the value of indexing EOA for PPM measurement has been discussed and re-evaluated, as it relates a measure of flow velocity to individual parameters two times (i.e., left ventricular outflow tract [LVOT] area and BSA) (13). In addition, based on a series of studies having measured Bernoulli's pressure gradients and patient-specific EOAs, Amorim et al. identified a significant correlation of transprosthetic pressure gradients to EOA. Thus, Amorim et al. claim that the use of the iEOA may be redundant and the use of transvalvular pressure gradients may be a practicable alternative option.

There is still a debate on whether measured or predicted iEOA is to prefer to correctly assess PPM following SAVR and TAVI. Predicted iEOA is based on various sources, such as mean echo data from various patient cohorts (14) or the reported size of manufacturers (15). Most studies which evaluated the impact of PPM on the outcome following SAVR used the predicted iEOA whereas more recent TAVI trials used the measured iEOA. The use of predicted iEOA re-classifies a certain proportion of patients toward a lower PPM grade, and the association to gradients and clinical outcomes are different (16).

The advantages and disadvantages of both parameters are listed in Table 2.

**Table 2:** Advantages and disadvantages of the use of measured and predicted PPM.

	**Measured PPM**	**Predicted PPM**
Accuracy	- Depends on echocardiographic quality (echo window, correctly obtained measurements, interobserver variability) - Depends on echocardiographic correctness (hemodynamic state of the patient, accounting for pressure recovery	Depends on the quality of the reference data
Association with clinical outcomes	Not consistent	Not consistent
Ease of use	Requires echocardiographic study of the patient; it is not clear which is the best time after SAVR or TAVR to assess PPM	Easy to use

For better comparability of clinical studies, a clear definition of PPM and a consistent recommendation whether the projected or measured iEOA should be used is required. A uniform recommendation for both TAVI and SAVR should be issued.

## Echocardiographic Assessment of PPM After TAVI and SAVR

Echocardiography remains the main imaging tool to assess the prosthesis function following TAVI and SAVR. As mentioned previously, PPM is characterized by the iEOA, which is the ratio of EOA and BSA. The thresholds as displayed in Table 1 are used for SAVR and TAVI.

**Figure 1:**
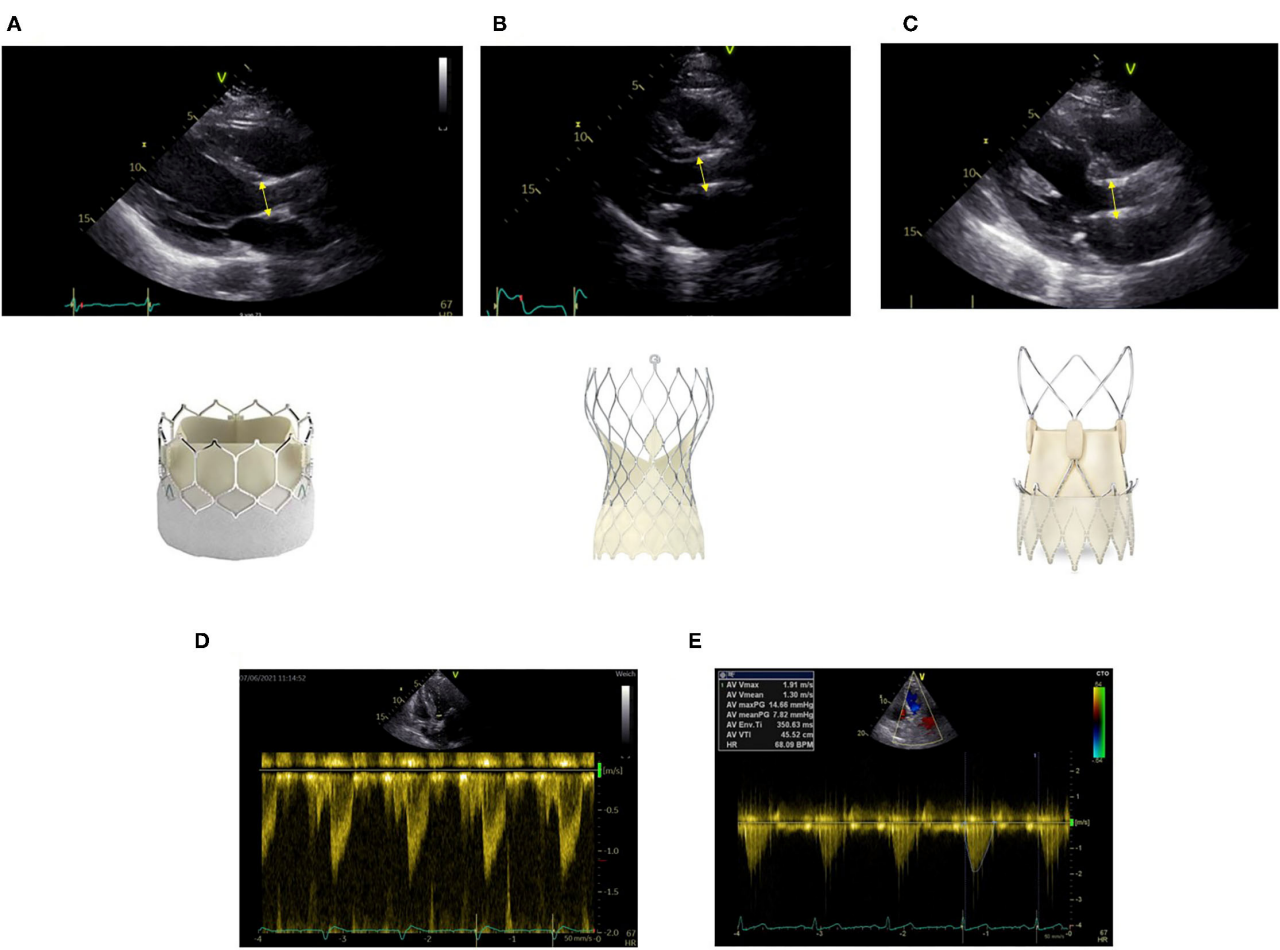
Correct measurement of left ventricular outflow tract (LVOT) diameter at the inferior edge of the stent as indicated by the yellow bar in different transcatheter aortic valve implantation (TAVI) prostheses **(A)** Sapien Ultra, **(B)** Evolut Pro, **(C)** Acurate neo2. Positioning of the pulse wave Doppler sample at the same level **(D)**. Obtainment of highest peak velocity by continuous wave Doppler **(E)**.

Due to the design of the transcatheter heart valves, there are two areas of flow acceleration: first at the level of the inferior edge of the stent and second at the level of the cusps. For correct measurement, it is crucial to measure LVOT diameter at the inferior edge of the stent (Figure 1A). It is important to measure from outer-edge to outer-edge. The different valve stents may challenge the echo-based LVOT measurements (Figures 1A–C). Echocardiographic measurements have to be made precisely. Potential measurement errors of the continuous and pulse wave Doppler signal need to be excluded (Figures 1D,E).

**Figure 2:**
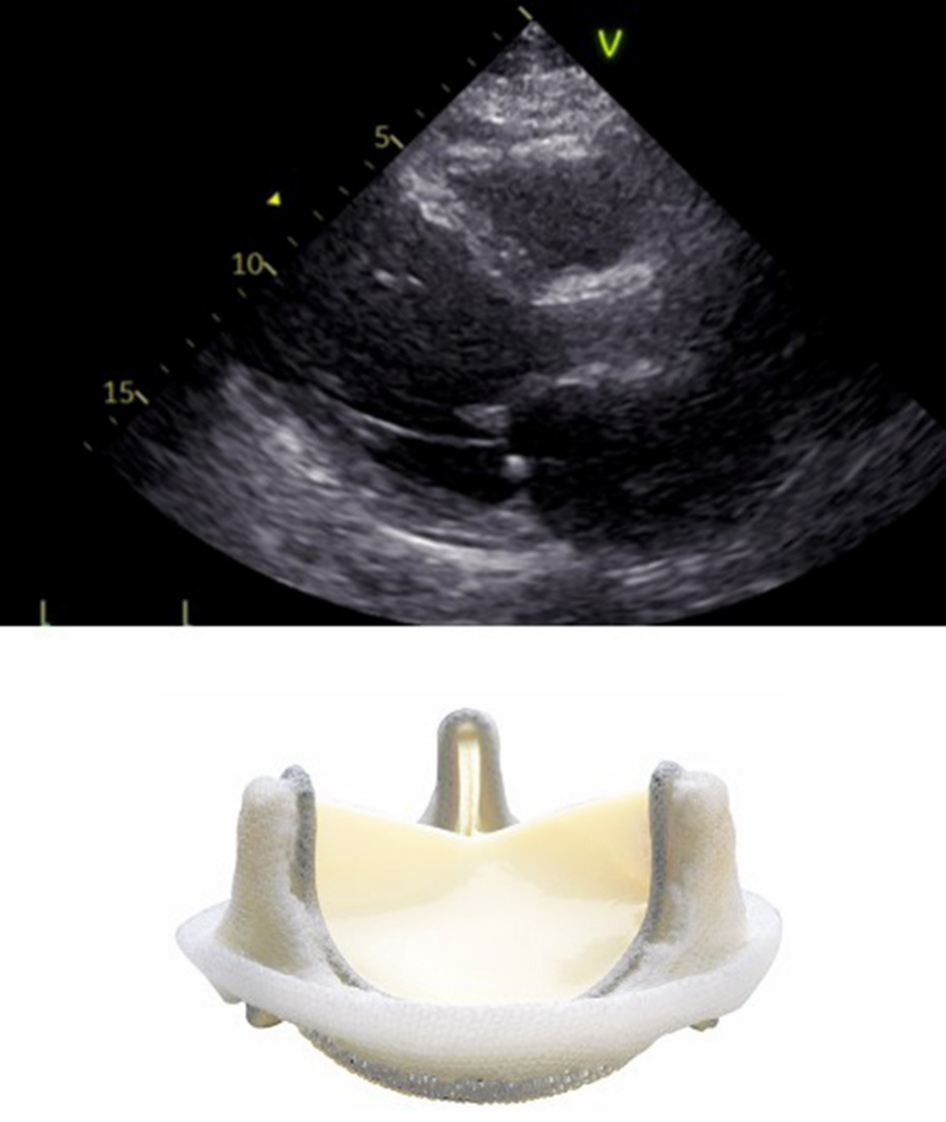
Correct measurement of LVOT diameter below the inferior edge of the suture ring as indicated by the yellow bar in a surgical bioprosthetic valve (Perimount 2900).

In surgical prostheses, there is only one area of flow acceleration within the suture ring. The LVOT should be measured outer-to outer edge at the inferior edge of the suture ring with the pulse wave Doppler sample positioned at the same level (Figure 2).

Measured iEOA is flow dependent and might be under- or overestimated in a low- or high-flow state. In particular, a low-flow state might result in a pseudo-severe PPM. In addition, depending on the aortic root anatomy, the echocardiographic cross-sectional transprosthetic jet area may differ more or less from the real area available for blood flow (13).

Pressure recovery is another important phenomenon which impacts Doppler derived gradients across the prosthetic valve. Due to deceleration of the blood flow between the aortic valve and the ascending aorta, kinetic energy is converted to static pressure thus increasing the transvalvular pressure gradient. Clinically, relevant pressure recovery occurs mainly in patients with small aortas (<30 mm) and should be considered while assessing hemodynamics of the prosthetic valve (12). As a corrective measure, the energy loss index (ELI) should be calculated and PPM adjusted to ELI accordingly. In a recent publication, in 1,217 patients following TAVI adjustment for pressure recovery revealed a significant proportion of patients who were reclassified. However, pressure recovery-adjusted PPM did not increase its association with cardiovascular mortality (17).

The phenomenon of pressure recovery explains the finding why Doppler derived gradients are usually higher than invasively measured transvalvular gradients (18) further underlining that echocardiographically-derived iEOA might be underestimating the real EOA and falsely classifying patients to have a relevant PPM.

The echocardiographically-based measurement of the LVOT, which enters the equation squared, is prone to erroneous measurements with a prosthetic valve in place. Due to reverberations and shadowing caused by the prosthesis exact measurement are sometimes challenging. In addition, the LVOT cross-section is usually elliptic and not circular as anticipated by the continuity equation. To overcome this issue, the CT-derived or three-dimensional transesophageal/transthoracic echocardiography (TOE/TTE)-derived LVOT diameter might be used instead. An analysis from the PARTNER2 Trial S3i cohort revealed indeed a significantly lower percentage of PPM when iEOA was calculated by using CT-derived LVOT measurements (19). However, for routine follow-up, the assessment of CT-based LVOT might not be practical. It is important to mention that the measured LVOT must not be substituted by the labeled size of the prosthesis (12).

If EOA cannot be determined due to insufficient imaging quality, the Doppler velocity index (DVI, calculated as ration of LVOT velocity time integral (VTI) to aortic VTI) can be used to assess the function of the prosthesis. In balloon-expandable valves, DVI should be >0.43 and self-expanding valve >0.59 (14).

During assessment of prosthetic valve dysfunction, it is important to distinguish PPM and other non-structural valve dysfunction from structural valve dysfunction, thrombosis and endocarditis. Here, clinical factors are useful to be taken into account. Reduced iEOA as a result of PPM is present immediately after valve implantation whereas later diagnosed reduced iEOA accompanied by increased transvalvular gradients usually indicates structural valve dysfunction. This dysfunction is due to permanent intrinsic changes of the prosthesis as, for example, either leaflet fibrosis, disruption, or flail, or strut fracture/deformation (20). Additional imaging may be useful to differentiate PPM from other types of valve dysfunction, such as 18F-GP1 PET to detect valve thrombosis (accepted, JIMG 2021, Bing et al.) Since the treatment options are completely different, a correct diagnosis is essential.

## Clinical Impact After SAVR

Numerous studies have investigated the association of PPM with clinical outcomes. Among those, the assessment of PPM and the determination of cut-off values was not uniform.

There are conflicting data, whether PPM affects early outcomes after SAVR, such as early mortality, renal failure, stroke, inotropic requirement, or prolonged ventilation (21, 22). It remains unclear, if the possible higher rate of postoperative complications is due to PPM itself or is simply a surrogate marker of comorbidity and a more complex patient (21).

The remaining higher gradient in patients with PPM may impede left ventricular mass regression after SAVR. Several studies have shown less complete left ventricular mass regression with higher degrees of PPM (10, 23–25), while others did not (26, 27). The same mechanism may predispose to faster degeneration of bioprosthetic valves following aortic valve replacement (28, 29).

As PPM may be seen as a residual stenosis after SAVR, patients may experience residual symptoms. Exercise studies revealed significantly higher mean aortic gradients on increasing exercise levels and lower percentage of the predicted VO2max achieved during exercise in patients with PPM (30, 31). However, it is very rare to perform reoperations for symptomatic PPM (32), because the predicted risk of a reintervention has to be balanced against the expected benefits.

The negative effects of a residual stenosis, e.g., incomplete left ventricular mass regression, or faster degeneration of bioprostheses may have an impact on long-term survival. A large meta-analysis including more that 27,000 patients found a significant impact of moderate and severe PPM on all-cause and cardiac-related survival beyond 5 years (33). Another meta-analysis including more than 40,000 patients found that the impact on mortality is more pronounced in patients <70 years, or with a body mass index (BMI) <28 kg/m^2^ (34). An age-dependent impact of PPM on longer term survival was also found by other groups (11, 24, 35).

## Impact of PPM After TAVI

Transcatheter aortic valve implantation introduced a new option and completely different approach for the treatment of aortic valve pathologies. As the native valve calcium is not removed but pushed aside, the potential area for transprosthetic valve flow may be limited. However, as opposed to SAVR prosthesis, TAVI prostheses consist only of a small stent frame instead of a bulkier sewing ring which might result in a larger area for transprosthetic valve flow with more favorable hemodynamics.

In the current literature, the incidence for PPM ranges from 24–48% for moderate and 8–18% for severe PPM (36). In the randomized PARTNER A cohort, moderate PPM was reported in 48% of the patients, whereas severe PPM occurred only in 19.7% of the patients (37). As opposed to the SAVR cohort PPM was neither an independent predictor for left ventricular mass regression nor for 2-year mortality in the TAVI cohort. Of note, PPM was an independent predictor of 1-year mortality in patients without post-procedural paravalvular leakage.

Since it is well appreciated from data on surgical prostheses that the risk for PPM is higher in a small aortic annulus anatomy, the TAVI-SMALL registry investigated in a retrospective analysis the incidence of PPM in various self-expanding TAVI valves (Evolut R, Evolut Pro, Acurate, and Portico) in 859 patients (38). Despite the retrospective design, baseline characteristics were well balanced between the groups. The rate of moderate PPM was significantly higher in the Portico group (38%) as compared with the other TAVI valves, which might be due to its intra-annular design in contrast to the supra-annular design of the other self-expanding valves. However, there was no difference in terms of severe PPM with an overall rate of 9.4%. In a subset of patients with a very small annulus, the incidence of severe PPM was slightly higher (13.7%) without any difference between the groups.

There is clear evidence that balloon-expandable valves are more prone for PPM as self-expanding valves due to their intra-annular design. The CHOICE extended registry showed a significantly higher rate of PPM for SAPIEN 3 (43.2%) as compared with Evolut R (21.7%) in patients with large as well as with small annuli (59.2 vs. 33.3%) (39).

A multicenter propensity-matched study (40) in 246 patients with an aortic annulus <400 mm^2^ undergoing TAVI with either the balloon-expandable SAPIEN 3 of the self-expanding ACURATE neo revealed a significantly higher rate of severe PPM in the balloon-expandable group (22 vs. 3%).

Apart from small aortic annulus, small LVOT and TAVI valve selection, PPM has been observed more often in patients with increased BMI (36, 37, 41, 42).

Whether PPM impacts prognosis following TAVI is still a matter of debate, as long-term results are missing and initial TAVI patients had multiple comorbidities. As mentioned above, PPM was predictive in the PARTNER A TAVI cohort when no paravalvular leakage was present (37).

Severe PPM was detected in 12.9% of the patients in a single-center registry (43) with a lower prevalence in self-expanding TAVI valves. In the overall cohort, PPM was not predictive for all-cause mortality, however, in patients with a reduced ejection fraction (EF < 40%), severe PPM was an independent factor of all-cause mortality after 3 years (hazard ratio [*HR*] 2.97; 95% *CI*: 1.58–5.59, *p* < 0.001). There was no impact on patients with EF > 40%.

Another single-center analysis revealed a 25% rate of severe PPM in the enrolled study cohort (44). Severe PPM had an independent predictive impact on event-free 3-year survival (52 vs. 84%, *p* = 0.04). There was no significant impact on stroke rate and rehospitalization for heart failure.

The multicenter WIN-TAVI registry (45), which exclusively enrolled female patients, reported a PPM rate of 32.8%. As described in other studies, in this cohort of female patients higher BMI and smaller TAVI prostheses were the only independent predictors of PPM. Of interest, PPM did not impact 1-year mortality or major cardiovascular event.

Moderate and severe PPM was observed in 8.9 and 0.7% of patients in the Ocean-TAVI trial (46), which included exclusively 1,546 Japanese patients. Multivariate analysis identified younger age, small aortic annulus complex, and implantation of a balloon-expandable valve as independent predictors. All-cause mortality was not different between patients with or without PPM (10.2 vs. 8.3%, *p* = 0.41).

From the current literature, there is some evidence that PPM impacts outcome in patients with reduced EF (43). Therefore, it should be consequently avoided in this patient group by selecting a self-expanding TAVI valve with supra-annular design. Since patients with small aortic annulus complex are more prone to PPM, valve selection should be made accordingly. The impact on survival in the overall TAVI population is still a matter of debate and further studies with a longer follow-up are required. As in surgical patients, PPM might have an impact on premature TAVI valve degeneration, however, this issue has not been investigated so far.

Table 3 provides an overview of the impact of measured and predicted PPM on the survival of large studies after SAVR and TAVI.

**Table 3:** Impact of PPM in major original studies with predicted or measured PPM.

**References**	***n***	**Measured or predicted EOA**	**Valve type**	**Follow-up**	**Patient age**	**PPM definition**	**PPM rate**	**Association of PPM with outcomes**
Rao et al. (47)	2,154	predicted	Different stented bioprostheses	74 ± 49 months	67 (PPM) 66 (no PPM)	iEOA ≤ 0.75 cm^2^/m^2^	10.5%	Early and late cardiovascular mortality
Moon et al. (35)	1,399	predicted	Different bioprostheses	46 ± 40 months	70 ± 13 (no PPM), 72 ± 12 (moderate PPM), 71 ± 12 (severe PPM)	Severe: iEOA <0.65 cm^2^/m^2^ Moderate: iEOA 0.65- ≤ 0.85 cm^2^/m^2^	Severe: 12.2% Moderate: 50.2%	negative impact on late survival for patients ≤ 70 years of age, but for patients >70 years of age, prosthesis–patient mismatch did not influence late survival
M ohty et al. (11)	2,576	predicted	Stented and stentless bioprostheses, mechanical prostheses	4.8 ± 3.4 years	68 ± 10 (no PPM), 71 ± 9 (moderate PPM), 69 ± 11 (severe PPM)	Severe: iEOA <0.65 cm^2^/m^2^ Moderate: iEOA 0.65- ≤ 0.85 cm^2^/m^2^	Severe: 2% Moderate: 31%	increase in late mortality with PPM only in patients <70 years old and/or with a BMI <30 kg/m^2^ or an LV ejection fraction <50%
Hong et al. (23)	351	measured	Stented bioprostheses and mechanical prostheses	12 years	60 ± 12 (no PPM), 59 ± 18 (mild PPM), 59 ± 13 (moderate PPM), 62 ± 14 (severe PPM)	Severe: iEOA <0.65 cm^2^/m^2^ Moderate: iEOA 0.65- ≤ 0.75 cm^2^/m^2^ Mild: 0.75- ≤ 0.85 cm^2^/m^2^	Severe: 10.3% Moderate: 11.1% Mild: 14.8%	Impact of severe PPM on long-term survival, and cardiac-related death; less LV mass regression
Bleiziffer et al. (10)	645	measured	Stented and stentless bioprostheses	Mean 2.66 years	72 ± 8 (no PPM), 72 ± 8 (PPM)	≤ 0.85 cm^2^/m^2^	moderate or severe: 39.9%	improved survival for larger iEOAs (iEOA as a continuous variable)
Pibarot et al. (37) (surgical cohort)	270	measured	Stented bioprostheses	1 year	84 ± 7 (no PPM), 85 ± 6 (PPM)	Severe: iEOA <0.65 cm^2^/m^2^ Moderate: iEOA 0.65- ≤ 0.85 cm^2^/m^2^	Severe: 28.1% Moderate: 31.9%	worse survival and less LV mass regression
Herrmann et al. (48)	62,125	measured	Different TAVI prostheses	1 year (analysis of 37,470 patients)	83 (no PPM), 81 (moderate PPM), 79 (severe PPM)	Severe: iEOA <0.65 cm^2^/m^2^ Moderate: iEOA 0.65– ≤ 0.85 cm^2^/m^2^	Severe: 12.1% Moderate: 24.6%	Higher mortality and heart failure rehospitalization at one year with severe PPM
Tang et al. (49)	47,620	measured	Supraannular TAVI prostheses	1 year		Severe: iEOA <0.65 cm^2^/m^2^ Moderate: iEOA 0.65– ≤ 0.85 cm^2^/m^2^	Severe: 5.3% (and 27% in valve-in-valve)	No association of severe PPM with mortality or valve-related readmissions
Schofer et al. (43)	1,309	measured	Different TAVI prostheses	2.03 years	81 ± 6 (no PPM), 81 ± 7 (moderate PPM), 80 ± 8 (severe PPM)	BMI <30 kg/m^2^: Severe: iEOA <0.65 cm^2^/m^2^ Moderate: iEOA <0.85 ≥0.65 cm/m^2^ BMI ≥30 kg/m^2^: Severe: iEOA <0.60 cm/m^2^ Moderate: iEOA <0.70 ≥0.60 cm/m^2^	Severe: 12.9% Moderate: 22.9%	increased all-cause mortality in EF <40% with severe PPM
Miyasaka et al. (46)	1,546	measured	Different TAVI prostheses		85 (82–88) (no PPM), 84 (80–87) (PPM)			No impact on all-cause mortality
Ternacle et al. (16)	1,088	Measured and predicted	Different TAVI prostheses	1 year	79.1 ± 8.4	BMI <30 kg/m^2^: Severe: iEOA ≤ 0.65 cm^2^/m^2^ Moderate: iEOA ≤ 0.85 >0.65 cm/m^2^ BMI ≥30 kg/m^2^: Severe: iEOA ≤ 0.55 cm/m^2^ Moderate: iEOA ± ≤ 0.70 ≥0.60 cm/m^2^	Severe: 1% (predicted = vs. 17% (measured) Moderate: 10% (predicted) vs. 27% (measured)	No association of clinical outcomes, stronger association of predicted PPM with hemodynamic outcomes

## PPM Prevention

### In Surgical Aortic Valve Replacement

Given the large body of literature showing a significant impact of PPM on clinical outcomes, there is consensus that PPM should be avoided at the time of operation (50). A number of clinical predictors are associated with the increased risk for PPM: older age, female sex, larger BSA and BMI, diabetes, hypertension, renal failure, and implantation of a bioprosthesis rather than a mechanical valve (34).

To choose an adequately sized prosthesis not only in the above-mentioned risk population, the iEOA has to be predicted prior to implantation of a certain prosthesis for the individual patient. In this context, it is important to understand that the observed EOA of a given prosthetic valve type and size varies from individual to individual and also within serial measurements in the same patient. The interindividual variation results mainly from different aortic root anatomies (13), while the intraindividual variation can be attributed to the flow status. Thus, the observed EOAs for a given type and size of normally functioning prostheses may show a wide range of values. The use of echocardiographically measured mean EOA values from preferably large numbers of patients helps to estimate and predict the iEOA of an individual patient prior to surgery. Such reference values can be extracted from many publications (4, 33), or are available at a smartphone application (CardioValve, Digimednet). Only reference tables based on echocardiographic measurements should be used (4).

If the required prosthesis size cannot be implanted in an individual anatomy, annular enlargement with patch augmentation can be performed. Additional surgical maneuvers should be considered preferably in younger patients and in those with left ventricular dysfunction, in whom the association of PPM with adverse clinical outcomes is most evident (21). There is no significant increase in surgical risk after adjustment for concomitant procedures with annular enlargement (51). Being more effective in increasing the EOA, the Manougian procedure should be preferred over the Nicks procedure (51).

In certain patients with small aortic anatomy, interventional treatment might be preferred over surgical aortic valve replacement and aortic root enlargement. In particular, older patients with favorable anatomy for TAVI might be good candidates for interventional treatment even though they exhibit only a low surgical risk. Thorough discussion in the heart valve team considering that shared-decision making is absolutely crucial under these circumstances.

### In TAVI

Reference values for the EOA of certain transcatheter heart valves are still limited (14), but should be used to predict the iEOA. As stated above, self-expanding TAVI valves with a supra-annular design (Acurate neo and Evolut R/Pro) have been shown to have lower transvalvular gradients and according to this higher measured iEOA. In patients with a small aortic area, the relative size of the stent and skirt may even reduce the potential opening area, while also in patients with a larger body size, the hemodynamic requirements should be taken into account (52). Based on the current data, the authors recommend to use a TAVI valve with supra-annular design in patients at risk for PPM. Additional preventive strategies, such as post-dilatation in case of an increased gradient or valve oversizing should be routinely implemented (53).

## Future Implications

Prosthesis-patient mismatch remains one out of many factors to be considered during decision-making for the treatment of aortic valve pathologies. The current ESC/EACTS guidelines for the management of patients with valvular heart disease 2021 include the recommendation to choose TAVI over SAVR in patients with expected PPM. The latest ACC/AHA guidelines 2020 contain the statement that TAVR provides a larger valve area than the same size SAVR, and that the option of annular enlargement should be taken into account when choosing the procedure and valve type (54).

Since there is still uncertainty whether predicted or measured iEOA should be used to assess PPM, both might be considered in a patient following SAVR and TAVI. Hereby, the limitations of measured iEOA needs to be taken into account in particular, flow status and potential overestimation of transvalvular gradient. Regarding predicted iEOA, its application is restricted due to the fact that reliable reference values for all currently implanted prosthesis—a dispensable pre-requisite—are not yet available.

Prosthesis-patient mismatch should be avoided to prevent sequelae of increased prosthetic gradients after aortic valve replacement. To prevent PPM, it is required to anticipate the iEOA of the prosthesis prior to the procedure. The use of adequate reference tables is most appropriate to predict the iEOA. Such tables should be provided soon also for all available transcatheter heart valves. As suggested by the joint EACTS–STS–AATS Valve Labeling Task Force (8), standardized valve charts could provide all essential information on the details of heart valve models. Such valve charts should also be introduced for transcatheter heart valve models.

During the decision-making process, all available options should be taken into account for the individual patient. If the predicted size and type of a surgical prosthesis cannot be implanted, additional surgical procedures, such as annular enlargement with the Manougian technique, or alternative procedures, such as TAVI can prevent PPM. According to the ESC/EACTS guidelines, TAVI may become the first line treatment for all patients with a small aortic root anatomy. To prevent PPM after TAVI, valve type and size selection, as well as procedural maneuvers, such as post-dilatation are of importance.

Prosthesis-patient mismatch seems to play a more significant role in younger patients—obviously because in an otherwise relatively healthy patient, PPM may become the only influencing factor for an unfavorable outcome (e.g., decreased exercise capacity due to higher gradients). With the shift of TAVI indications toward lower risk and younger patients, the prediction and avoidance of PPM will gain relevance during individual patient assessment prior to the procedure. The PARTNER data have already shown that PPM was an independent predictor of 1-year mortality in patients with TAVI without post-procedural paravalvular leakage.

Future studies on the topic of PPM should harmonize the PPM assessment methods among TAVR and SAVR. The establishment of reliable reference values for all available prosthesis types for assessment and prediction of PPM is desirable. Uniform iEOA thresholds of 0.65 and 0.85 cm^2^/m^2^ for severe and moderate mismatch in normal weight patients, 0.55 and 0.7 cm^2^/m^2^ for adipose patients with BMI ≥ 30 kg/m^2^ should be used in all studies. The adherence to this practice improves comparability of scientific studies and increases the quality of clinical assessments during follow-up after TAVI and SAVR.

## Data Availability Statement

The original contributions presented in the study are included in the article/supplementary material, further inquiries can be directed to the corresponding author.

## Author Contributions

SB prepared the concept and structure of the manuscript. SB and TR equally contributed to the sections of the manuscript. All authors contributed to manuscript revision, read, and approved the submitted version.

## Conflict of Interest

The authors declare that the research was conducted in the absence of any commercial or financial relationships that could be construed as a potential conflict of interest.

## Publisher's Note

All claims expressed in this article are solely those of the authors and do not necessarily represent those of their affiliated organizations, or those of the publisher, the editors and the reviewers. Any product that may be evaluated in this article, or claim that may be made by its manufacturer, is not guaranteed or endorsed by the publisher.
